# RNA-Seq reveals differential expression profiles and functional annotation of genes involved in retinal degeneration in Pde6c mutant *Danio rerio*

**DOI:** 10.1186/s12864-020-6550-z

**Published:** 2020-02-07

**Authors:** Madhu Sudhana Saddala, Anton Lennikov, Adam Bouras, Hu Huang

**Affiliations:** 1 School of Medicine, Department Ophthalmology, Mason Eye Institute, University of Missouri-Columbia, One Hospital Drive, MA102C, Columbia, MO 65212 USA; 2 Wilmer Eye Institute, Johns Hopkins University, Baltimore, MD USA

**Keywords:** Pde6c, Zebrafish, Gene ontology, FastQC, Trinity, KEGG

## Abstract

**Background:**

Retinal degenerative diseases affect millions of people and represent the leading cause of vision loss around the world. Retinal degeneration has been attributed to a wide variety of causes, such as disruption of genes involved in phototransduction, biosynthesis, folding of the rhodopsin molecule, and the structural support of the retina. The molecular pathogenesis of the biological events in retinal degeneration is unclear; however, the molecular basis of the retinal pathological defect can be potentially determined by gene-expression profiling of the whole retina. In the present study, we analyzed the differential gene expression profile of the retina from a wild-type zebrafish and phosphodiesterase 6c (*pde6c*) mutant.

**Results:**

The datasets were downloaded from the Sequence Read Archive (SRA), and adaptors and unbiased bases were removed, and sequences were checked to ensure the quality. The reads were further aligned to the reference genome of zebrafish, and the gene expression was calculated. The differentially expressed genes (DEGs) were filtered based on the log fold change (logFC) (±4) and *p*-values (*p* < 0.001). We performed gene annotation (molecular function [MF], biological process [BP], cellular component [CC]), and determined the functional pathways Kyoto Encyclopedia of Genes and Genomes (KEGG) pathway for the DEGs. Our result showed 216 upregulated and 3527 downregulated genes between normal and pde6c mutant zebrafish. These DEGs are involved in various KEGG pathways, such as the phototransduction (12 genes), mRNA surveillance (17 genes), phagosome (25 genes), glycolysis/gluconeogenesis (15 genes), adrenergic signaling in cardiomyocytes (29 genes), ribosome (20 genes), the citrate cycle (TCA cycle; 8 genes), insulin signaling (24 genes), oxidative phosphorylation (20 genes), and RNA transport (22 genes) pathways. Many more of all the pathway genes were down-regulated, while fewer were up-regulated in the retina of pde6c mutant zebrafish.

**Conclusions:**

Our data strongly indicate that, among these genes, the above-mentioned pathways’ genes as well as calcium-binding, neural damage, peptidase, immunological, and apoptosis proteins are mostly involved in the retinal and neural degeneration that cause abnormalities in photoreceptors or retinal pigment epithelium (RPE) cells.

## Background

Retinal degeneration is retinopathy that consists of the deterioration of the retina due to the progressive death of its cells [1]. It is a common cause of blindness, and it can result from mutations in a large variety of structural and enzymatic proteins of the photoreceptors [2]. Degenerative diseases of the retina, including retinitis pigmentosa (RP), affect nearly 2 million patients worldwide [3]. A wide variety of causes have been attributed to retinal degeneration, such as disruption of the genes involved in phototransduction, biosynthesis, folding of the rhodopsin molecule, and the structural support of the retina [4]. In zebrafish mutants, an A > G point mutation was identified in the pde6c (phosphodiesterase 6C, cyclic guanosine monophosphate [cGMP]-specific, cone, alpha prime) gene [5]. This gene encodes the beta subunit of the phosphodiesterase 6 (*Pde6*) protein, which is essential for the proper functioning of the photoreceptor cells [6]. The beta subunit is one of two catalytic subunits of the pde6 protein, which combines with two inhibitory gamma subunits to form the effector enzyme of rod phototransduction [7]. Light stimulation triggers a cascade of reactions, leading to the hydrolysis of cGMP by pde6, and the resulting change in cGMP concentration directly alters the membrane channels to produce the electrical response of the photoreceptors [7]. Due to the high cooperativity of cGMP binding to the channel [8], a small increase in cGMP levels will have a profound effect on the number of open channels and the cations (Na^+^, Ca^2+^) that pass through them. Humans harboring loss-of-function PDE6b mutations develop RP, progressing to total blindness as a function of age [9, 10]. This mutation has been predicted to cause a frameshift in the coding sequence and result either in a truncated pde6c or degradation of pde6c mRNA through nonsense-mediated decay; it ultimately affects both cone and rod photoreceptors [7]. Human PDE6c mutations have been reported and linked to autosomal recessive achromatopsia [11, 12]. Moreover, the PDE6c mutant zebrafish was introduced as a model organism that recapitulates many properties of human PDE6c patients [7]. These animals develop rapid photoreceptor cell loss that progresses with age and is followed by complete loss of visual functions. Understanding which genes are perturbed in the photoreceptor degeneration could pave the way for the identification of biomarkers as potential predictors of disease onset, as well as elucidating the pathways involved in the degenerative process, as the zebrafish as a model organism that allows rapid screening of a multitude of substances and therapeutic approaches.

In this study, we used publicly available pde6c mutant and wild type zebrafish retina whole transcriptome shotgun sequencing (RNA-Seq) datasets [13] to examine differentially expressed genes (DEGs), gene ontology (GO), and functional pathway analysis. We seek to characterize the signal pathways and genes that are potentially involved in retinal degeneration in general and photoreceptor degeneration in particular, as well as to better understand the molecular mechanisms that underlie the retinal degenerative disorders by using transcriptomic and bioinformatics approaches.

## Results

### Differential gene expression analysis

**Fig. 1:**
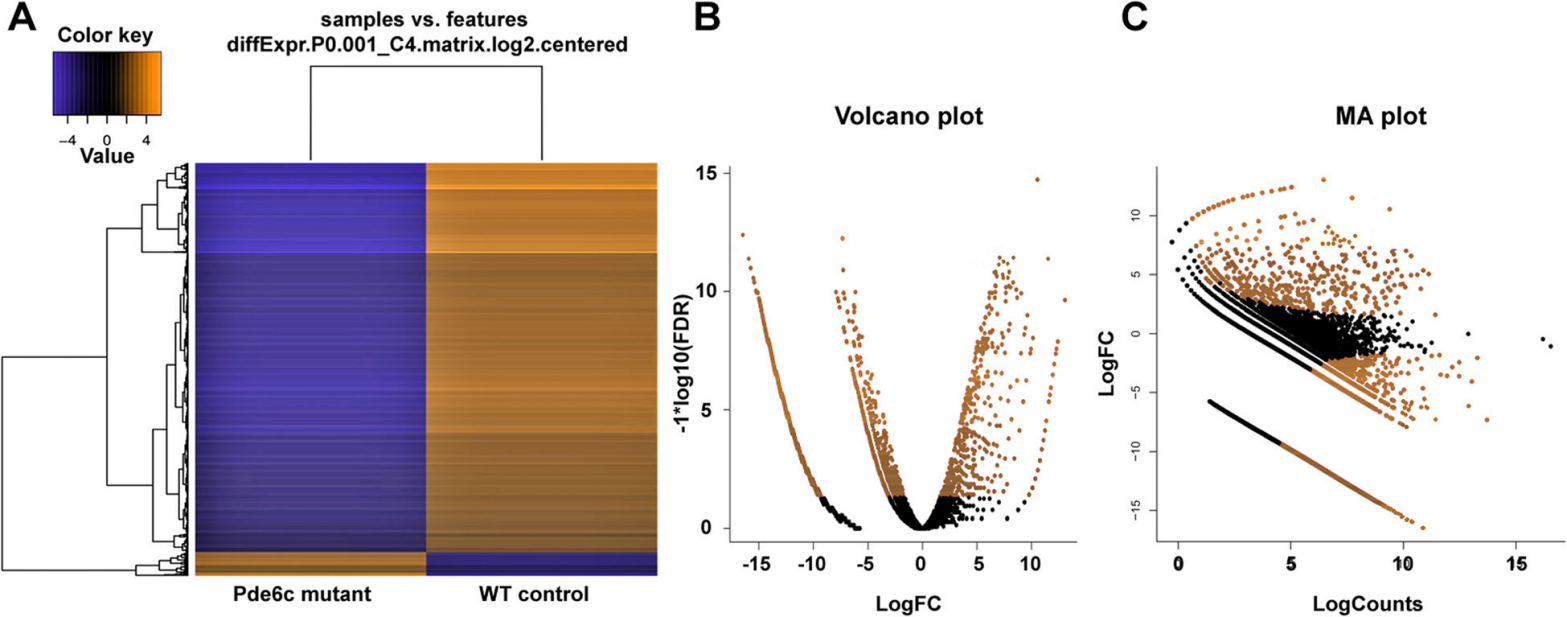
Heatmap, volcano and MA plots. **a** The heatmap of up- and down-regulated genes in orange and blue, respectively. **b** The volcano plot was constructed by plotting the negative log of the log10 FDR value on the y-axis. This results in data points with low log10 FDR values (highly significant) appearing toward the top of the plot. The x-axis is the logFC between the two conditions (wild and mutant zebrafish). **c** MA plot visualizes the differences between measurements taken in wild and mutant zebrafish DEGs, by transforming the data into M (log ratio) and A (mean average) scales logCPM (counts per million) and logFC, then plotting these values. The orange color indicates the significant genes, and the black color indicates non-significant genes

**Fig. 2:**
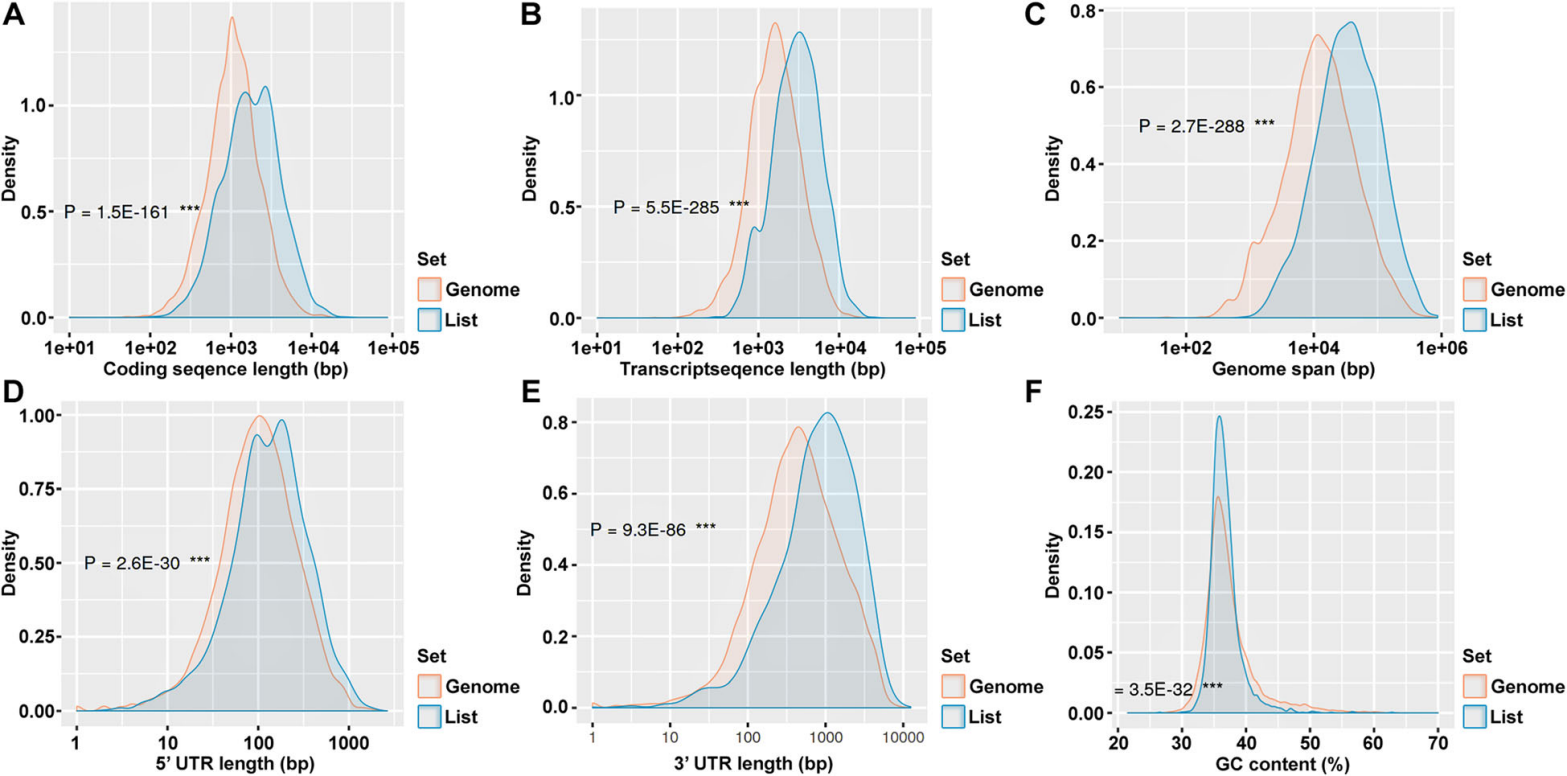
The gene density. **a** coding sequence length base pair (bp), **b** transcript length (bp), **c** genome span (bp), **d** 5′ untranslated region (UTR) length (bp), **e** 3′ UTR length (bp), **f** and percentage of the GC (Guanine, Cytocine) content of DEGs

**Fig. 3:**
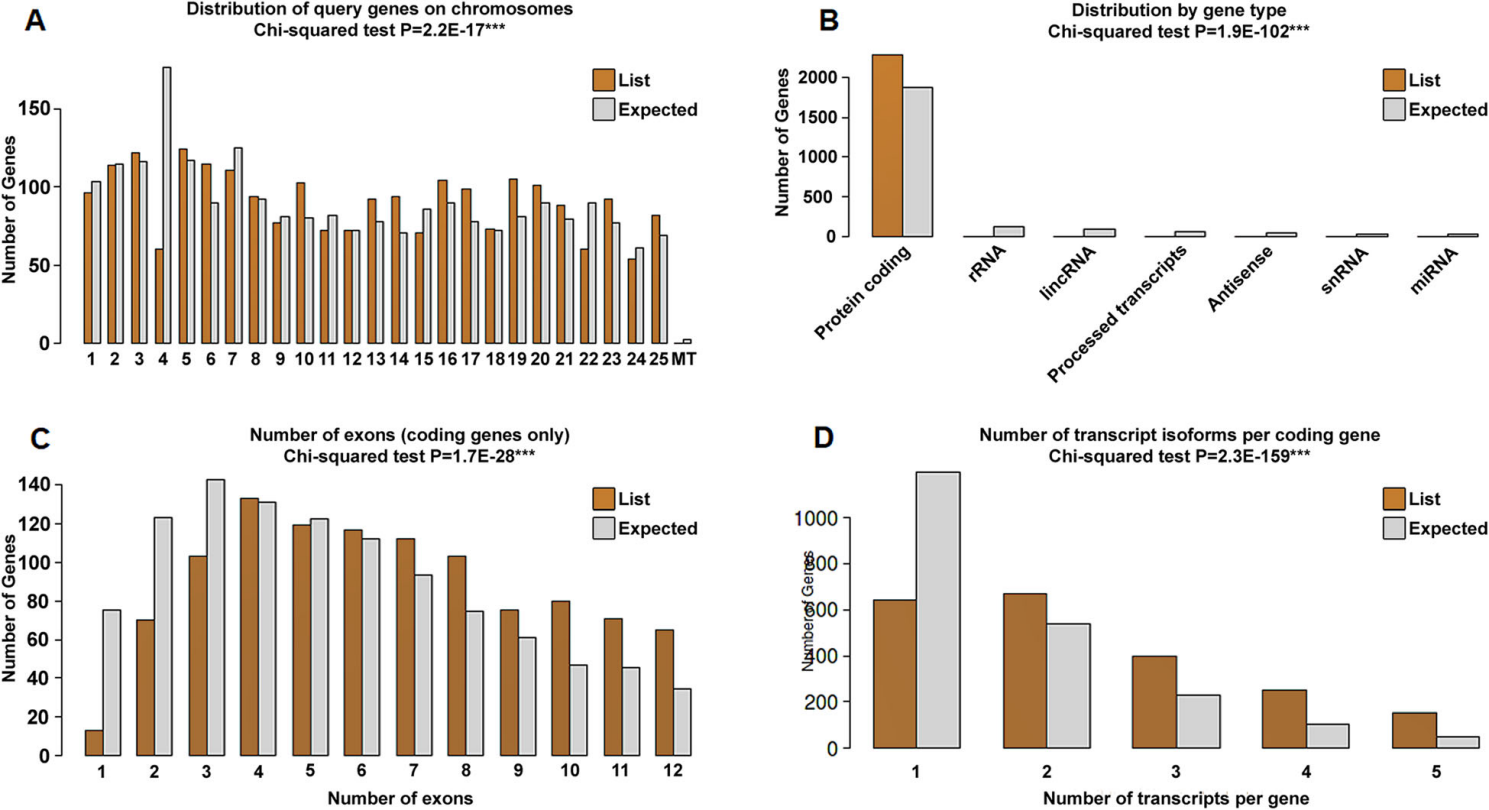
Genome-wide distribution of DEGs on zebrafish chromosomes. **a** Distribution of query genes across 25 chromosomes of zebrafish and mitochondria genome. **b** Distribution by gene type. **c** Distribution of genes through the exons. **d** Number of transcripts isoforms per coding gene

We discovered 216 up-regulated and 3527 down-regulated DEGs in the pde6c mutant conditions. The hierarchical clustering heatmap, MA plot, and volcano plots were generated to represent the up- and down-regulated genes (logFC ±4 and *p* < 0.001). Figure 1a represents the heatmap of up- and down-regulated genes in orange and blue, respectively. The volcano plot (Fig. 1b) and the MA plot (Fig. 1c) visualizes the differences between measurements taken in wild and mutant zebrafish DEGs. The gene density is presented in Fig. 2, demonstrating all parts of the genes, such as the coding sequence length, transcript length, genome span, 5′ UTR length, 3′ UTR length, and percentage of GC content compared with the zebrafish genome’s density. The results revealed that all the parts of the gene’s density (List, DEGs) fluctuate compared with the zebrafish genome. Also, we predicted the distribution of DEGs on zebrafish chromosomes (genome-wide distribution), distribution of gene type, number of exons (coding genes), and number of transcript isoforms per coding gene. Figure 3a revealed that all the query genes were distributed on 25 chromosomes, and the mitochondria genome of zebrafish with the exception of chromosome 4. Figure 3b shows that the protein-coding (mRNA) was more distributed than the others, while Fig. 3c shows that exon 4 is more distributed among the number of genes, and Fig. 3d shows that one and two transcripts per gene are equally distributed among the number of genes.

### Functional annotation

**Fig. 4:**
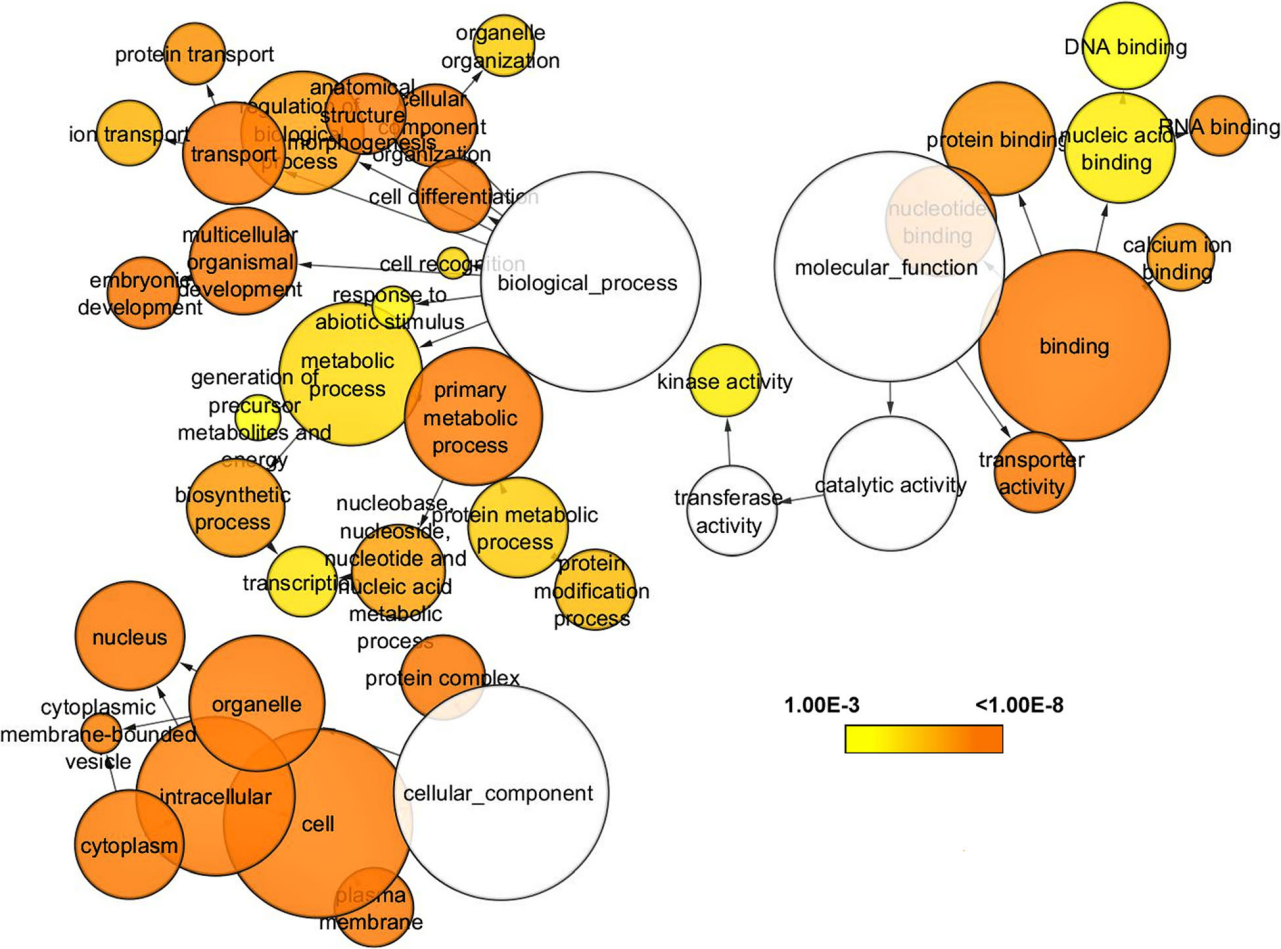
Gene Ontology enrichment analysis like biological process (BP), cellular component (CC) and molecular functions (MF)

All the DEGs were uploaded to the GO Enrichment Analysis tool and database for annotation, visualization and integrated discovery (DAVID) tool using the complete zebrafish genome as the background. The molecular functions (MFs), biological processes (BPs), cellular components (CCs), and pathways were predicted in the significantly enriched GO terms of the differentially express genes (Fig. 4). The DEGs were involved in various MFs, such as small molecule binding (GO: 0036094; FDR = 6.33e− 11), nucleotide-binding (GO: 0000166; FDR = 6.52e− 11), nucleoside phosphate binding (GO: 1901265; FDR = 6.52e− 11), cation-transporting ATPase activity (GO: 0019829; FDR = 1.34e− 08), ATPase-coupled ion transmembrane transporter activity (GO: 0042625; FDR = 1.34e− 08), active ion transmembrane transporter activity (GO: 0022853; FDR = 2.33e− 08), purine nucleotide binding (GO: 0017076; FDR = 2.43e− 08), purine ribonucleotide binding (GO: 0032555; FDR = 3.78e− 08), nucleoside binding (GO: 0001882; FDR = 3.78e− 08), and ribonucleotide binding (GO: 0032553; FDR = 4.45e− 08) functions. Among these MFs, most of the genes are involved in small molecule binding (171 genes), and nucleoside phosphate binding (164 genes) functions.

The DEGs are involved in various BPs, such as embryo development (GO: 0009790; FDR = 5.81e− 11), retina development in camera-type eyes (GO: 0060041; FDR = 2.48e− 10), system development (GO: 0048731; FDR = 2.48e− 10), embryo development ending in birth or egg hatching (GO: 0009792; FDR = 2.48e− 10), chordate embryonic development (GO: 0043009; FDR = 2.48e− 10), animal organ development (GO: 0048513; FDR = 6.93e− 10), eye development (GO: 0001654; FDR = 2.44e− 09), camera-type eye development (GO: 0043010; FDR = 5.81e− 09), sensory organ development (GO:0007423; FDR = 1.62e− 07), and the cellular developmental process (GO: 0048869; FDR = 9e− 07). Among these BPs, most genes are involved in system development (184 genes), animal organ development (141 genes), and cellular developmental processes (121 genes).

The DEGs are involved in various CCs, such as the macromolecular complex (GO: 0032991; FDR = 0e+ 00), cytosol (GO: 0005829; FDR = 2.18e− 12), cell periphery (GO: 0071944; FDR = 1.61e− 11), plasma membrane (GO: 0005886; FDR = 1.61e− 11), protein complex (GO: 0043234; FDR = 1.61e− 11), membrane protein complex (GO: 0098796; FDR = 9.62e− 11), neuron part (GO: 0097458; FDR = 3.74e− 10), plasma membrane part (GO: 0044459; FDR = 3.84e− 10), whole membrane (GO: 0098805; FDR = 5.66e− 10), and non-membrane-bounded organelle (GO: 0043228; FDR = 1.24e− 08) functions. Most genes are involved in the macromolecular complex (169 genes), cell periphery (107 genes), and plasma membrane (105 genes).

### Pathway analysis

**Fig. 5:**
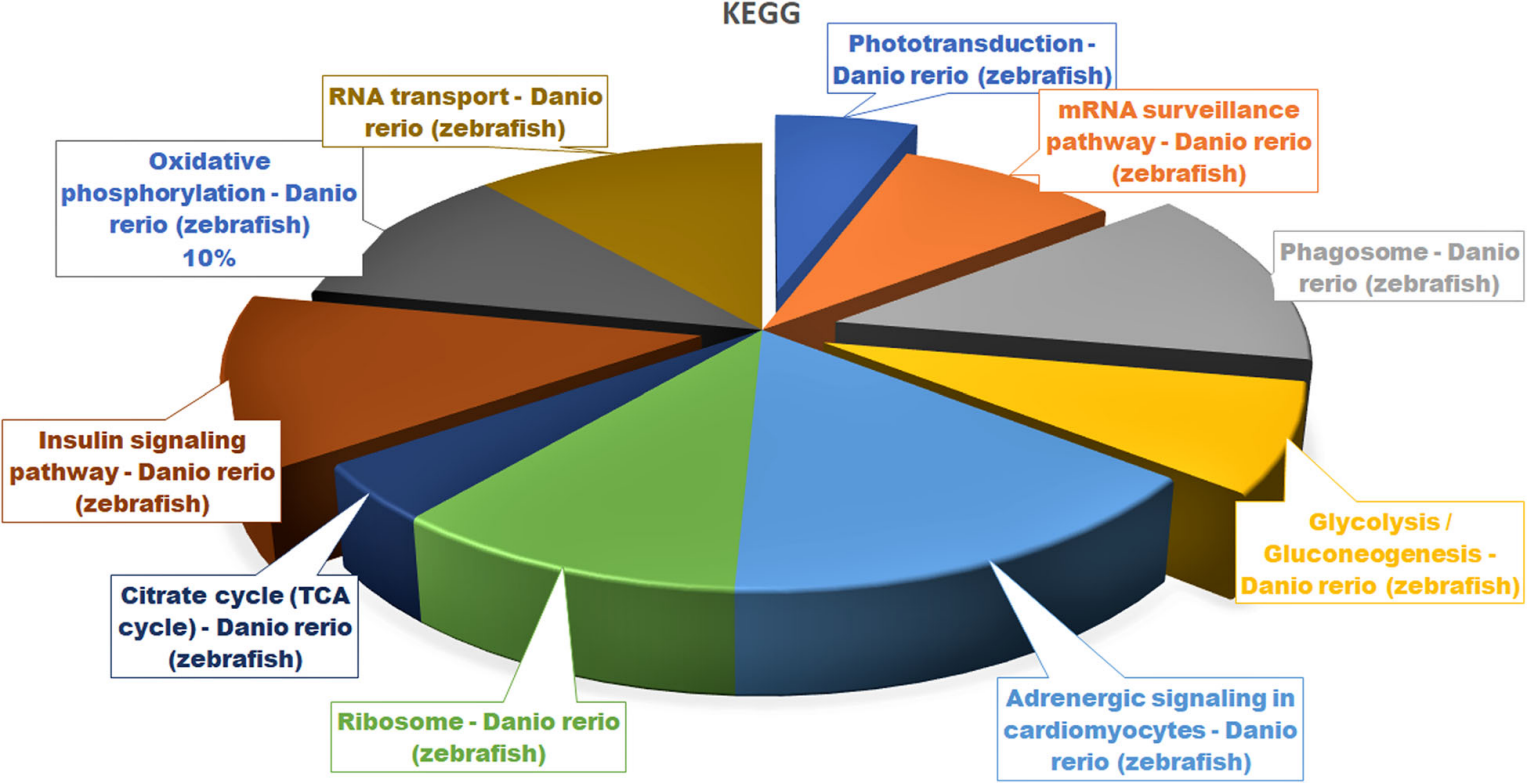
Functional pathway enrichment analysis. The DEGs are involved in various KEGG biological pathways

Pathway analysis helps elucidate data from canonical prior knowledge structured in the form of pathways. It allows finding distinct cell processes, diseases, or signaling pathways that are statistically associated with the selection of DEGs [14]. The DEGs are further analyzed in the pathway functional analysis using the DAVID annotation tool (Fig. 5). They are involved in various KEGG pathways, such as the phototransduction (12 genes), mRNA surveillance (17 genes), phagosome (25 genes), glycolysis/gluconeogenesis (15 genes), adrenergic signaling in cardiomyocytes (29 genes), ribosome (20 genes), citrate cycle (TCA cycle; 8 genes), insulin signaling (24 genes), oxidative phosphorylation (20 genes), and RNA transport (22 genes) pathways. Most genes are involved in adrenergic signaling in the cardiomyocyte (29 genes), phagosome (25 genes), insulin signaling (24 genes), and RNA transport pathways (20 genes).

In this study, we focus on the phototransduction (dre04744; Table 1), phagosome (dre04145; Table 2), glycolysis/gluconeogenesis (dre00010; Table 3) and insulin signaling pathways (dre04910; Table 4).

**Table 1:** List of genes involved in Phototransduction pathway of Pde6c mutant zebrafish (*p*-value = 0.0014000; FDR = 0.0039000)

Ref mRNA	Gene Symbol	Gene Name	logFC	*p*-value
NM_001007160	pde6a	phosphodiesterase 6A, cGMP-specific, rod, alpha	−12.88482348	9.29E-08
NM_001017711	grk1b	G protein-coupled receptor kinase 1 b	−15.21450566	1.36E-13
NM_001030248	rcvrna	recoverin a	−14.7635283	1.67E-12
NM_001031841	grk7a	G protein-coupled receptor kinase 7a	−14.51580863	8.55E-12
NM_001034181	grk1a	G protein-coupled receptor kinase 1 a	−5.203467963	2.80E-06
NM_001327800	rgs9a	regulator of G protein signaling 9a	−5.215468297	1.45E-08
NM_131084	rho	rhodopsin	−6.296723464	8.77E-13
NM_131868	gnat1	guanine nucleotide binding protein (G protein), alpha transducing activity polypeptide 1	−7.218093348	2.15E-13
NM_131869	gnat2	guanine nucleotide binding protein (G protein), alpha transducing activity polypeptide 2	−4.074631105	4.60E-08
NM_199570	calm3b	calmodulin 3b (phosphorylase kinase, delta)	−14.42999547	1.49E-11
NM_199996	calm2a	calmodulin 2a (phosphorylase kinase, delta)	−13.04988312	3.41E-08
NM_213481	gnb1b	guanine nucleotide binding protein (G protein), beta polypeptide 1b	−13.12286971	2.17E-08

**Table 2:** List of genes involved in phagosome pathway of pde6c mutant zebrafish (*p*-value = 5.68e-04; FDR = 2.6e-02)

Ref mRNA	Gene Symbol	Gene Name	logFC	*p*-value
NM_001033721	itgav	integrin, alpha V	−11.90966981	8.44E-06
NM_153659	sec61a1	Sec61 translocon alpha 1 subunit	−4.751020073	2.47E-07
NM_173254	atp6v1e1b	ATPase, H+ transporting, lysosomal, V1 subunit E1b	−5.280581114	1.80E-06
NM_173255	atp6v0ca	ATPase, H+ transporting, lysosomal, V0 subunit ca	−13.63501996	1.50E-09
NM_199561	atp6v0b	ATPase, H+ transporting, lysosomal V0 subunit b	−12.22047943	1.52E-06
NM_199620	atp6v0d1	ATPase, H+ transporting, lysosomal V0 subunit d1	−12.91329746	7.59E-08
NM_199713	calr	calreticulin	−11.48042737	4.49E-05
NM_199934	atp6v1g1	ATPase, H+ transporting, lysosomal, V1 subunit G1	−12.06864677	3.55E-06
NM_201485	rab5aa	RAB5A, member RAS oncogene family, a	−12.76579558	1.10E-07
NM_201322	atp6v1c1a	ATPase, H+ transporting, lysosomal, V1 subunit C1a	−12.8196416	7.94E-08
NM_194388	tuba1b	tubulin, alpha 1b	−11.23913699	8.52E-05
NM_198818	tubb5	tubulin, beta 5	−4.073625492	4.71E-05
NM_200414	cybb	cytochrome b-245, beta polypeptide (chronic granulomatous disease)	5.802066863	3.87E-12
NM_201135	atp6v1aa	ATPase, H+ transporting, lysosomal, V1 subunit Aa	−13.31681197	6.58E-09
NM_213030	tuba2	tubulin, alpha 2	−12.88637707	8.98E-08
NM_213448	canx	calnexin	−12.86210644	6.21E-08
NM_001002526	atp6v1f	ATPase, H+ transporting, lysosomal, V1 subunit F	−11.68530135	1.63E-05
NM_001005772	atp6v1c1b	ATPase, H+ transporting, lysosomal, V1 subunit C1b	−11.19719131	0.000111091
NM_001020666	atp6v0a1b	ATPase, H+ transporting, lysosomal V0 subunit a1b	−13.28947797	7.71E-09
NM_001172635	atpv0e2	ATPase, H+ transporting V0 subunit e2	−12.58243475	3.26E-07
NM_001082836	itgb5	integrin, beta 5	−11.19217538	0.000111091
NM_001105126	tuba1c	tubulin, alpha 1c	−14.61096727	4.55E-12
NM_131031	actb1	actin, beta 1	−5.667990423	5.34E-09
NM_181601	actb2	actin, beta 2	−4.641227854	5.35E-09
NM_001037410	tubb2b	tubulin, beta 2b	−5.70469418	6.49E-10

**Table 3:** List of genes involved in glycolysis/gluconeogenesis pathway of Pde6c mutant zebrafish (*p*-value = 6.8e-04; FDR = 2.6e-02)

Ref mRNA	Gene Symbol	Gene Name	logFC	*p*-value
NM_153667	tpi1a	triosephosphate isomerase 1a	−12.15496243	2.18E-06
NM_131246	ldha	lactate dehydrogenase A4	−13.07189634	2.92E-08
NM_131247	ldhba	lactate dehydrogenase Ba	−13.11162313	2.30E-08
NM_212667	dlat	dihydrolipoamide S-acetyltransferase (E2 component of pyruvate dehydrogenase complex)	−11.98410994	5.58E-06
NM_201300	pgam1b	phosphoglycerate mutase 1b	−5.097181249	5.05E-06
NM_212724	aldh7a1	aldehyde dehydrogenase 7 family, member A1	−13.09492311	2.59E-08
NM_194377	aldoaa	aldolase a, fructose-bisphosphate, a	−11.6637211	1.75E-05
NM_214751	pck1	phosphoenolpyruvate carboxykinase 1 (soluble)	5.129531889	7.55E-09
NM_201506	dldh	dihydrolipoamide dehydrogenase	−12.15238215	2.18E-06
NM_213094	gapdhs	glyceraldehyde-3-phosphate dehydrogenase, spermatogenic	−5.832123465	3.72E-11
NM_213387	pgk1	phosphoglycerate kinase 1	−12.67163046	1.94E-07
NM_213252	hk1	hexokinase 1	−13.41102831	3.67E-09
NM_001328389	pfklb	phosphofructokinase, liver b	−11.29868758	6.58E-05
NM_153668	tpi1b	triosephosphate isomerase 1b	−13.58805684	2.03E-09
NM_001080066	g6pc3	glucose 6 phosphatase, catalytic, 3	4.465924297	9.85E-08

**Table 4:** List of genes involved in insulin signalling pathway of Pde6c mutant zebrafish (*p*-value = 5.64e-03; FDR = 9.83e-02)

Ref mRNA	Gene Symbol	Gene Name	logFC	*p*-value
NM_001004527	ppp1cb	protein phosphatase 1, catalytic subunit, beta isozyme	−13.3391067	5.63E-09
NM_131855	prkci	protein kinase C, iota	−11.4865862	4.49E-05
NM_213075	flot2a	flotillin 2a	−11.94140373	7.05E-06
NM_001142672	insra	insulin receptor a	−4.775105577	2.21E-05
NM_001123229	insrb	insulin receptor b	−11.53691235	3.33E-05
NM_131381	gsk3b	glycogen synthase kinase 3 beta	−11.65098191	1.87E-05
NM_199570	calm3b	calmodulin 3b (phosphorylase kinase, delta)	−14.42999547	1.49E-11
NM_001077211	mtor	mechanistic target of rapamycin (serine/threonine kinase)	−12.74710586	1.23E-07
NM_212710	ppp1cab	protein phosphatase 1, catalytic subunit, alpha isozyme b	−4.868811067	1.31E-05
NM_199996	calm2a	calmodulin2a (p hosphorylase kinase, delta)	−13.04988312	3.41E-08
NM_194402	mknk2b	MAP kinase interacting serine/threonine kinase 2b	−14.02356489	1.26E-10
NM_214751	pck1	phosphoenolpyruvate carboxykinase 1 (soluble)	5.129531889	7.55E-09
NM_200315	irs2a	insulin receptor substrate 2a	−12.51373347	4.75E-07
NM_201023	prkacba	protein kinase, cAMP-dependent, catalytic, beta a	4.638063399	9.62E-09
NM_205744	braf	B-Raf proto-oncogene, serine/threonine kinase	−11.88966935	5.38E-06
NM_213035	grb2b	growth factor receptor-bound protein 2b	−12.43630299	7.66E-07
NM_213076	rps6kb1b	ribosomal protein S6 kinase b, polypeptide 1b	−11.31030831	6.05E-05
NM_213161	prkag1	protein kinase, AMP-activated, gamma 1 non-catalytic subunit	−11.20468266	0.000101616
NM_213252	hk1	hexokinase 1	−13.41102831	3.67E-09
NM_001017732	prkar1ab	protein kinase, cAMP-dependent, regulatory, type I, alpha (tissue specific extinguisher 1) b	−11.53493265	3.59E-05
NM_001077370	prkar2aa	protein kinase, cAMP-dependent, regulatory, type II, alpha A	−4.330637539	1.19E-05
NM_001281844	pik3r1	phosphoinositide-3-kinase, regulatory subunit 1 (alpha)	−13.92572365	2.37E-10
NM_131721	mapk8b	mitogen-activated protein kinase 8b	−5.355743879	1.18E-06
NM_001080066	g6pc3	glucose 6 phosphatase, catalytic, 3	4.465924297	9.85E-08

### Gene network analysis

**Fig. 6:**
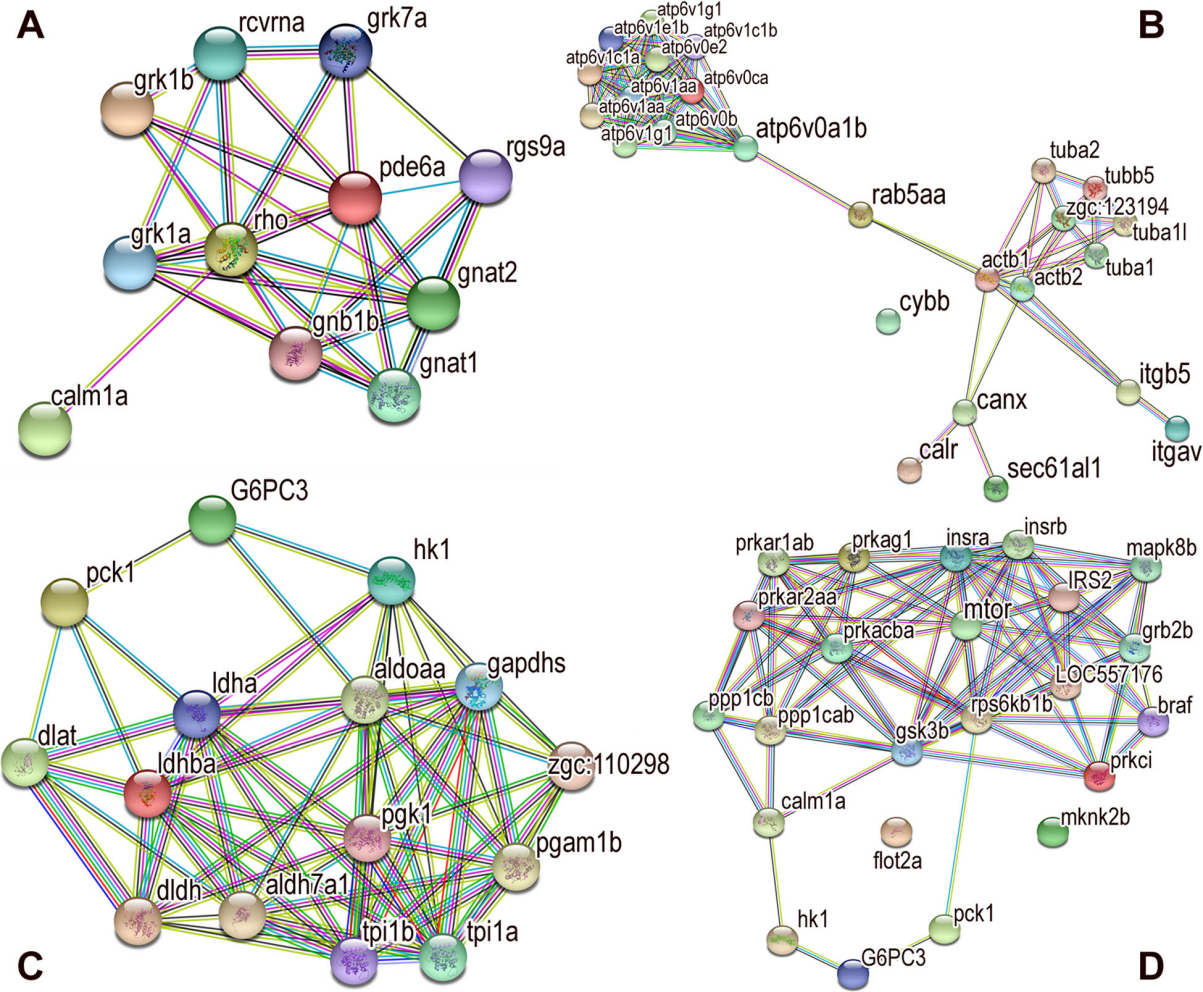
The gene network analysis and interaction subnetworks. **a** Phototransduction pathway; **b** The phagosome pathway; **c** The glycolysis/gluconeogenesis pathway; D The insulin signaling pathway subnetwork

The DEGs were used to construct gene-gene interactions using the STRING tool (https://string-db.org/), which also hides the disconnected nodes in the network. The results showed the analyzed number of nodes (426), expected number of edges (1235), the number of edges (1512), average node degree (7.1), average local clustering coefficient (0.363), and Protein-protein interaction (PPI) enrichment *p* < 1.58e− 14. We constructed the gene-gene network for DEGs with their respective minimum required interaction score (0.400). We mapped the phagosome (red), glycolysis/gluconeogenesis (blue), and insulin signaling (green) pathway genes’ interaction in the global network (Additional file 1: Figure S1). These pathways genes interactions are presented individually as subnetworks. The phototransduction pathway subnetwork showed the number of nodes as 11, expected number of edges as 1, real number of edges as 32, average node degree as 5.82, average local clustering coefficient as 0.743, and PPI enrichment as *p* < 1.0e− 16. The subnetwork results suggested that all the genes involved were directly connected and involved in the phototransduction pathway (Fig. 6a). The phagosome pathway genes’ subnetwork results showed the number of nodes as 25, expected number of edges as 16, real number of edges as 83, average node degree as 6.64, average local clustering coefficient as 0.803, and PPI enrichment as *p* < 1.0e− 16. This subnetwork genes’ interaction results showed that the cybb (cytochrome b-245 beta) gene did not interact with any genes, but the remaining genes connected directly or indirectly to each other. This gene (cybb) may be involved individually in the phagosome pathway (Fig. 6b). The glycolysis/gluconeogenesis pathway subnetwork showed the number of nodes as 15, expected number of edges as 3, real number of edges as 65, average node degree as 8.67, average local clustering coefficient as 0.741, and PPI enrichment as *p* < 1.0e− 16. These subnetwork results suggested that all the genes involved were directly connected and involved in the glycolysis/gluconeogenesis pathway (Fig. 6c). The insulin signaling pathway subnetwork showed the number of nodes as 23, expected number of edges as 44, the real number of edges as 87, average node degree as 5.57, average local clustering coefficient as 0.585, and PPI enrichment as *p* < 6.47e− 09. These subnetwork results suggested that the flot2a (flotillin-2a) and mknk2b (MAPK interacting serine/threonine kinase 2b) genes are not connected to any genes, but the other genes are connected directly or indirectly (Fig. 6d). The flot2a and mknk2b genes are involved in the insulin signaling pathway individually.

## Discussion

We provide a comprehensive transcriptomic analysis of wild and mutant zebrafish retina datasets. This approach may provide a gene expression profile for the wild-type and pdec6c mutant zebrafish retinal models. Mapping the genes and its expression values to the heatmap, volcano and MA plots demonstrated clear separation between wild-type and pdec6c mutant zebrafish with the predominance of the down-regulated genes in the latter indicating it’s a crucial role in the retinal cells function.

The pathway enrichment analysis and gene-gene network analysis revealed that the DEGs are involved in various KEGG functional pathways, such as the phototransduction, phagosome, glycolysis/gluconeogenesis, and insulin signaling pathways. Twelve genes are involved in the phototransduction pathway and down-regulated in pde6c mutant zebrafish. Zhang et al. [13] reported the role of the phototransduction pathway genes in retinal degeneration. Stearns et al. [7] described how the mutation of the pde6 gene causes rapid cone photoreceptor degeneration in the zebrafish model. Our results also strongly correlated with the above-lighted findings.

Seventeen genes are involved in the phagosome pathway and down-regulated in the pde6c mutant. These genes interact with each other and are involved in retinal degeneration. Among these genes, the v-ATPase gene is essential for secretion, lysosome function, vesicular traffic, and phagocytosis [15]. In the zebrafish eye, V-ATPase regulates retinoblastoma proliferation and survival, possibly through the acidification resulting from proton accumulation [16]. The same H^+^ proton pump is essential for the activation of Wnt, JNK, and Notch signaling by regulating endosomal pH [17]. As per the above observations, biophysical and molecular approaches were used to address the ion nature and respective ion transporters involved in regeneration in an adult vertebrate (zebrafish). Our results suggested that V-ATPase is down-regulated, and these ATPases may be involved in the retinal degeneration in mutant zebrafish.

Fifteen genes are involved in the glycolysis/gluconeogenesis pathway, and they are down-regulated in mutant zebrafish except for the g6pc3 gene, which is up-regulated. It is a central pathway that produces important precursor metabolites, namely, six-carbon compounds of glucose-6P and fructose-6P and three-carbon compounds of glycerone-P, glyceraldehyde-3P, glycerate-3P, phosphoenolpyruvate, and pyruvate [18]. Gluconeogenesis is a synthesis pathway of glucose from noncarbohydrate precursors. It is essentially a reversal of glycolysis, with minor variations of alternative paths [19]. Glucose 6 phosphatase dehydrogenase (G6PDH) activity is regulated by the NADP^+^/NADPH ratio; NADPH inhibits its activity, whereas NADP^+^ is required for its proper active conformation [20]. In the non-oxidative part of the pentose phosphate pathway (PPP), Ru5P is converted into ribose-5-phosphate (R5P) by ribulose-5-phosphate isomerase (RPIA), and R5P may re-enter the glycolytic pathway when converted into fructose-6-phosphate (F6P) or glyceraldehyde-3-phosphate (G3P) [21]. Increased flux of glucose through the pentose phosphate pathways can have a neuroprotective function [22]. All the glycolysis/gluconeogenesis genes are downregulated in the mutant zebrafish and might be involved in the retinal degeneration mechanism.

Twenty-four genes are involved in insulin signaling pathways, and they are down-regulated in mutant zebrafish. Our results showed that all the insulin signaling pathway genes are downregulated in the mutant zebrafish and may be involved in the retinal degeneration mechanism. Meanwhile, other pathways are also down-regulated, including pyruvate metabolism, oxidative phosphorylation, TCA cycle pathways, pyruvate metabolic processes, and proton-transporting ATP synthase complexes, which reflects a decrease in the need for mitochondrial oxidative capacity in dedifferentiating cells. This is analogous to the processes of somatic and oncogenic cellular reprogramming to a pluripotent state, in which reprogrammed cells undergo metabolic “rewiring” that reduces both mitochondrial content and oxidative phosphorylation capacity [23].

The detailed description of the up-regulated genes presented in the Additional file 1. Here, 18 notable up-regulated genes that might be most related to the retinal degenerative process. Calhm2 (calcium homeostasis modulator family member 2) is activated by membrane depolarization, although its kinetic responses are distinct [24]. Calhm2 and connexins have similar structural features that confer both shared and distinct functional properties. They act as a sensor of extracellular Ca^2+^ in the brain; they may also participate in similar signaling functions in the retina [25]. The tnmd (tenomodulin) gene increases VEGF-A (vascular endothelial growth factor A) production, initiates the VEGFR (vascular endothelial growth factor receptor) signaling pathway, and leads to enhanced angiogenesis [26]. Rosenthal et al. suggested that the fgf1b (fibroblast growth factor 1b) gene increases the L-type Ca^2+^ channel [27] activity of retinal pigment epithelium (RPE) cells, resulting in an increase of VEGF-A secretion from RPE cells [28]. Yun et al. [29, 30] also proposed that elevated TNF (Tumor necrosis factor) levels have been associated with different autoimmune diseases, and deregulation of tnfrsf1a (TNF receptor superfamily member 1A) expression and signaling can lead to chronic inflammation and tissue damage. The role of the klf1 (kruppel like factor 1) gene in zebrafish comprises hematopoiesis, blood vessel function, and fin and epidermal development [31]. The pla2g12a (Phospholipase A2 Group 12A) gene is up-regulated in inflammation and atherosclerosis [32]. Deblandre et al. [33] suggested that neurl2 (neuralized-like protein 2) interacts with XDelta1 (xenopus delta1) and regulates Notch signaling. This signaling is involved in pathologic angiogenesis [34, 35], which is associated with tumor growth and ischemic stroke [36]. Ding et al. [37] suggested that phosphorylation of the plek (pleckstrin) gene increases proinflammatory cytokine secretion by mononuclear phagocytes in diabetes mellitus. The skap1 (src kinase associated phosphoprotein 1) gene plays a role in physiological retinal angiogenesis and the pathogenesis of retinal neovascularization [38]. The scgn (secretagogin) gene is a secthe reted calcium-binding protein found in the cytoplasm. It is related to calbindin D-28 K and calretinin. This protein is thought to be involved in potassium chloride (KCL)-stimulated calcium flux and cell proliferation [39]. Deangelis et al. [40] suggested that the HtrA Serine Peptidase 1 gene alters the risk of neovascular age-related macular degeneration. The tspan13a (tetraspanin 13a) gene defects affecting this protein cause a variety of progressive retinal degenerations in humans and mice and illustrate its importance for the formation and long-term stability of outer photoreceptor segments [41]. Xu et al. [23] suggested that lysosomal tspan13a gene is associated with retinal degeneration. The casp6 (caspase-6) gene is involved in neuronal apoptosis and the regenerative failure of injured retinal ganglion cells [42]. Ratnayaka et al. [43] proposed that app (amyloid beta) is involved in retinal degeneration. Overall, the gene network results suggested that most of the pathway genes interacted directly or indirectly with each other and were involved in specific cascade signaling pathways in retinal degeneration.

Our data strongly indicate that, among these genes, calcium-binding proteins, neural damage proteins, peptidase proteins, immunological proteins, and apoptosis proteins are mostly involved in retinal and neural degeneration.

Study limitations:

1. The small sample size of three *Pde6c* mutant and three control zebrafish retina datasets (Table 5) is a limitation of the current study, while this number is still sufficient for the useful analysis more substantial cohort of samples will allow identifying the genes that were not detected as DEGs in the current work.
2. While the nature of the mutant *Pde6c* gene restricts its effect to photoreceptor cells, however, we cannot definitively exclude unknown expression or roles of pde6c in other cell types as the mutation is global and not tissue specific.

**Table 5:** Detailed information of datasets of zebrafish (pde6c mutant and wildtype)

Bio Sample	Sample name	MBytes	zebrafish
SRR5833546	SAMN07358654	8838	pde6c mutant
SRR5833545	SAMN07358655	8774	pde6c mutant
SRR5833544	SAMN07358656	9229	pde6c mutant
SRR5833543	SAMN07358652	9321	wildtype
SRR5833542	SAMN07358653	8084	wildtype
SRR5833541	SAMN07358651	9611	wildtype

## Conclusion

In conclusion, the gene expression studies in eye tissue are an initial step in determining functions for putatively associated retinal and neural degeneration risk genes. The RNA-Seq transcriptome data analysis showed the gene expression profile between mutant and wild-type zebrafish models of retinal degeneration. The analysis of mutant versus wild-type zebrafish retina data gives insight into potential genes and pathways that may be targeted in future therapeutic studies and expands the knowledge of the progression of retinal degeneration.

## Methods

### Data quality and preprocessing

The RNA-Seq paired-end zebrafish (*Danio rerio*) wild and pde6c mutant retina data (SRP112616) were acquired from the National Centre for Biotechnology Information—Sequence Read Archive (NCBI-SRA; http://www.ncbi.nlm.nih.gov/sra) using the SRA Toolkit (https://www.ncbi.nlm.nih.gov/sra/docs/toolkitsoft/) with a prefetch function, save for one file (SRR5833546). The three paired SRA files (each group) were converted into fastq files (six files) with fastq-dump and split-files functions (Table 5). Initially, we performed a visualization of the quality of all datasets before and after trimming the adaptors and going through the preprocessing steps by using the FastQC tool (https://www.bioinformatics.babraham.ac.uk/projects/fastqc/) [44]. Finally, we removed any low-quality reads by trimming the bases from the 3′ and 5′ ends and maintaining a Phred-score ≤ 30 using the Trimmomatic-0.36 tool [45]. After cleaning and trimming of low-quality reads and adaptor removal, more than 96% of good quality reads in each stage were retained. These cleaned reads were used for further transcriptome assembly analysis.

### Reference-based assembly

All the datasets were assembled separately with a reference genome (zebrafish) using Bowtie 1.2.2 software [46]. Initially, Bowtie makes an indexes of genome file and align short reads to reference genome (Dani rerio: GRCz11,2017). Then, RSEM (RNA-Seq by Expectation-Maximization) [47] was used to estimate the number of RNA-Seq fragments that map to each contig with gene annotations in a GTF file. Because the abundance of individual transcripts may significantly differ between samples, the reads from each sample must be examined separately, resulting in sample-specific abundance values.

### Identification of differentially expressed genes (DEGs)

The Bioconductor tool was used with the edgeR package to analyze differential expression analysis in the assembled transcriptome [48, 49]. The wild and Pde6c mutant *Danio rerio* comparison transcript counts (matrix file) were used for differential gene expression with the Empirical Analysis of Digital Gene Expression in R (edgeR) package of Bioconductor with primary parameters like the false discovery rate (FDR), log fold change (logFC), log counts per million (logCPM), and *p*-value [48, 50]. Unigenes with *p*-values less than 0.001 (*p* < 0.001) and fold change of more than 4 (logFC > 4) were considered significantly differentially expressed. The differentially expressed genes were visualized by volcano plot, MA plot and heatmaps respectively. A volcano plot is a type of scatterplot that is used to quickly identify changes in large datasets composed of replicate data. A heatmap is a graphical representation of data that uses a color-coding system to represent different values. It combines a measure of statistical significance from a statistical test (*p*-value from an analysis of variance [ANOVA] model) with the magnitude of the change, enabling quick visual identification of those data points (genes) that display large magnitude changes that are also statistically significant [51]. An MA plot is an application of a Bland–Altman plot for a visual representation of genomic data [52]. The log2 fold change for a particular comparison is plotted on the y-axis and the average of the counts normalized by size factor is shown on the x-axis (“M” for minus, because a log-ratio is equal to log minus log, and “A” for average).

### Functional annotation

Gene ontology (GO) Enrichment Analysis (http://geneontology.org/page/go-enrichment-analysis) and DAVID annotation (https://david.ncifcrf.gov/) was used for functional annotation and pathway analysis, such as for the MFs, BPs, and CCs and KEGG pathways. GO terms with FDR (q < 0.05) were considered significantly enriched within the gene set [53, 54].

### Gene network analysis

We performed protein-protein network analysis for all the DEGs using the STRING 10.5 database (https://string-db.org/), a useful tool for understanding metabolic pathways, and predicting or developing genotype-phenotype associations [55].

### Statistical analysis

All the numeric values were expressed as the mean ± standard deviation (SD) of the respective groups. Statistical analyses were performed using Trinity software (https://github.com/trinityrnaseq/trinityrnaseq/wiki). Student *t*-tests and Benjamini–Hochberg corrections (FDR) were used. A *p*-value of less than 0.001 was considered significant.

## Supplementary information

**Additional file 1: FigureS1**. Gene-gene interaction network of all differentially expressed genes. We mapped the phagosome (red), glycolysis/gluconeogenesis (blue), and insulin signaling (green) pathway genes’ interaction in the global network.

## Data Availability

The datasets SRP112616 analyzed during the current study are available in the National Centre for Biotechnology Information—Sequence Read Archive (NCBI-SRA) repository, https://www.ncbi.nlm.nih.gov/sra/?term=SRP112616. All data generated or analyzed during this study are included in this published article and its supplementary information files. The raw files of the analysis pipeline output and information on the analysis pipeline can be provided on reasonable request to the corresponding author.

## Abbreviations

APP: Amyloid beta
BP: Biological process
CALHM2: Calcium homeostasis modulator family member 2
CASP6: Caspase-6
CC: Cellular component
cGMP: Cyclic guanosine monophosphate
CYBB: Cytochrome b-245 beta
DAVID: Database for annotation, visualization, and integrated discovery
DEGs: Differentially expressed genes
edgeR: Empirical analysis of Digital Gene Expression in R
F6P: Fructose-6-phosphate
FDR: False discovery rate
FGF1B: Fibroblast growth factor 1b
FLOT2A: Flotillin-2a
G3P: Glyceraldehyde-3-phosphate
G6PDH: Glucose 6 phosphatase dehydrogenase
GO: Gene ontology
KCL: Potassium chloride
KEGG: Kyoto Encyclopedia of Genes and Genomes
KLF1: Kruppel like factor 1
logCPM: Log counts per million
logFC: Log fold change
MF: Molecular function
MKNK2B: MAPK interacting serine/threonine kinase 2b
NCBI: National Centre for Biotechnology Information
NEURL2: Neuralized-like protein 2
PDE6C: Phosphodiesterase 6c
PLA2G12A: Phospholipase A2 Group 12A
PLEK: Pleckstrin
R5P: Ribose-5-phosphate
RP: Retinitis pigmentosa
RPE: Retinal pigment epithelium
RSEM: RNA-Seq by Expectation-Maximization
SCGN: Secretagogin
SD: Standard deviation
SKAP1: Src kinase associated phosphoprotein 1
SRA: Sequence Read Archive
TNF: Tumour necrosis factor
TNFRSF1A: TNF receptor superfamily member 1A
TSPAN13A: Tetraspanin 13a
VEGF-A: Vascular endothelial growth factor-A
VEGFR: Vascular endothelial growth factor receptor
XD1: Xenopus delta1
