# Supplementary material for: RNA-Seq reveals differential expression profiles and functional annotation of genes involved in retinal degeneration in Pde6c mutant Danio rerio
Source: BMC Genomics. 2020 Feb 7;21:132. doi: 10.1186/s12864-020-6550-z (PMC7006399; doi:10.1186/s12864-020-6550-z)
Supplement: Supplementary file 1 — Additional file 1: FigureS1. Gene-gene interaction network of all differentially expressed genes. We mapped the phagosome (red), glycolysis/gluconeogenesis (blue), and insulin signaling (green) pathway genes’ interaction in the global network. [file 12864_2020_6550_MOESM1_ESM.docx]

**RNA-Seq reveals differential expression profiles and functional annotation of genes involved in retinal degeneration in Pde6c mutant *Danio rerio: Supplementary Data***

Madhu Sudhana Saddala^1,2^, Anton Lennikov^1,2^, Adam Bouras^1^, Hu Huang^1,2^*

^1^ Mason Eye Institute, University of Missouri, Columbia, Missouri, United States of America

^2^Wilmer Eye Institute, Johns Hopkins University, Baltimore, Maryland, United States of America

*Corresponding author: Hu Huang, PhD


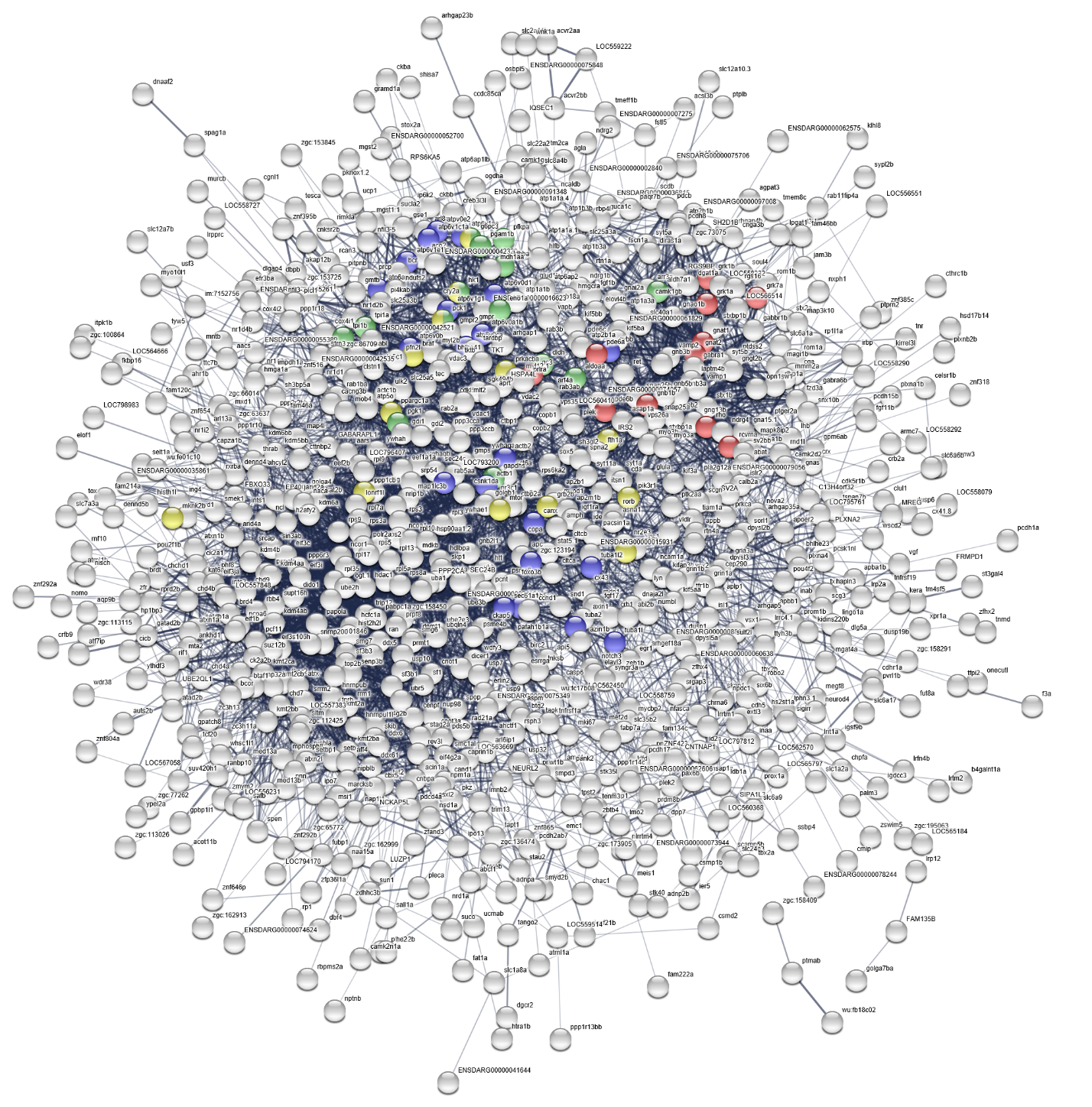
 **Figure S1:** Phagosome (red), glycolysis/gluconeogenesis (blue), and insulin signaling (green) pathway genes’ interactions.
